# Enhancement of the Rates for Insertion of Zinc(II) Ions into a Cationic Porphyrin Catalyzed by Poly(glutamate)

**DOI:** 10.3390/ijms242417371

**Published:** 2023-12-12

**Authors:** Roberto Zagami, Maria Angela Castriciano, Andrea Romeo, Luigi Monsù Scolaro

**Affiliations:** Department of Chemical, Biological, Pharmaceutical and Environmental Sciences, University of Messina, V.le F. Stagno D’Alcontres, 31, 98166 Messina, Italy; rzagami@unime.it (R.Z.); mcastriciano@unime.it (M.A.C.); lmonsu@unime.it (L.M.S.)

**Keywords:** porphyrins, self-assembled systems, kinetics, polyelectrolytes

## Abstract

The self-assembly of porphyrins onto polyelectrolytes could lead to interesting changes in their reactivity with respect to the bulk solution. Here, we investigated the kinetics of Zn^2+^ incorporation into tetra-cationic water-soluble 5,10,15,20-tetrakis-(N-methylpyridinium-4-yl)porphyrin (TMpyP(4)) in the presence of poly(L-glutamic acid) (PGA) in a pH range from 4 to 6.5. Under these conditions, the porphyrin electrostatically interacted with the polymer, which gradually switched from an α-helical to a random coil structure. The profile of the logarithm of the observed rate constant (*k_obs_*) versus the pH was sigmoidal with an inflection point close to the pH of the conformation transition for PGA. At a pH of 5.4, when PGA was in its highly charged random coil conformation, an almost 1000-fold increase in the reaction rates was observed. An increase in the ionic strength of the bulk solution led to a decrease in the metal insertion rates. The role of the charged matrix was explained in terms of its ability to assemble both reagents in proximity, in agreement with the theory of counter-ion condensation around polyelectrolytes in an aqueous solution.

## 1. Introduction

The generally accepted mechanism for metal ion coordination into the central core of porphyrins is based on the rate-determining step formation of a highly debated *sitting-atop* (SAT) complex. In this intermediate, the metal ion sits on top of the porphyrin plane, preceding the eventual expulsion of two protons and the formation of the metalloporphyrin [1,2,3,4]. The majority of the kinetic investigations were performed in the presence of salts [5,6] or other ligands (e.g., pyridines or imidazole derivatives [7,8]), sometimes revealing their catalytic role in the specific reaction. Only a few kinetic studies have addressed self-aggregated porphyrins [9,10], or porphyrins interacting with charged polymers. Pasternack et al. reported a strong deceleration of the metalation rates exerted by DNA on the metalation of the cationic porphyrins 5,10,15,20-tetrakis-(N-methylpyridinium-4-yl)porphyrin (TMpyP(4)) and 5,10,15,20-tetrakis-(4-N-trimethylaminophenyl)porphyrin (TAPP) [11]. Pioneering studies by Morawetz demonstrated that polyvinyl-sulfonate was very effective in accelerating the reaction rates of bound metal complexes, exhibiting a 10^5^-fold increase with respect to the absence of polymers [12]. According to the polyelectrolyte theory, charged polymers are able to enormously enhance the concentration of species bearing opposite charges in their immediate surroundings, thus increasing the number of reactive encounters [13]. On the contrary, these kinds of polymers have the tendency to inhibit reactions between oppositely charged species due to their repulsive effect on ions bearing charges of the same sign [14].

The ability of charged polymeric or complex matrices to accelerate the incorporation of metal ions in porphyrins has been exploited for the design of specific supramolecular sensing systems. Previous investigations have pointed out the efficacy of poly(glutamate) in strongly enhancing zinc(II) and copper(II) insertion into the core of TMpyP(4), thus facilitating the fast, quantitative detection of these ions in solution [15]. Quite recently, a series of supramolecular systems based on DNA fragments [16] or charged graphene quantum dots and porphyrins [17] have been proposed for the selective determination of various metal ions.

**Scheme 1:**
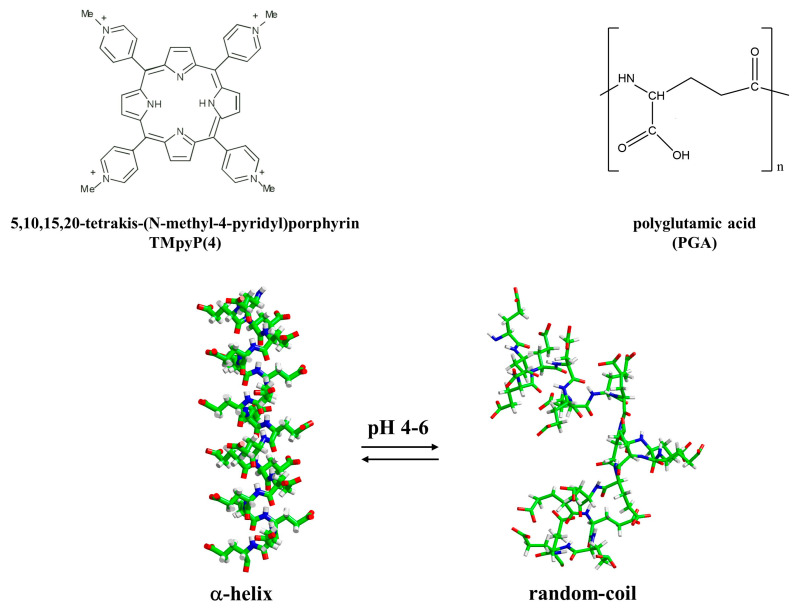
Molecular structure of the porphyrin TMpyP(4) and PGA, together with a representation of the pH-induced transition from an α-helix to a random coil.

On these bases, here we investigated in detail the kinetics of zinc(II) metal ion coordination into TMpyP(4) porphyrins when they are interacting with poly(L-glutamic acid) (PGA) (Scheme 1). PGA is a nice model system that, upon protonation, switches from a highly negatively charged random coil conformation to a less charged and stiff α-helical structure [18,19]. The choice of Zn^2+^ was dictated by the possibility of using a simple mixing procedure, thus avoiding the stopped-flow technique required, e.g., by the more reactive Cu^2+^ ions. Other metal ions were not considered because of their much higher inertia [2]. We anticipate that the tetracationic TMpyP(4) coordinates Zn^2+^ metal ions with an almost 1000-fold increase in its reaction rates in comparison to bulk solutions. In addition, the observed enhancement followed a sigmoidal profile that was reminiscent of the conformational changes in the PGA polymer.

## 2. Results and Discussion

**Figure 1:**
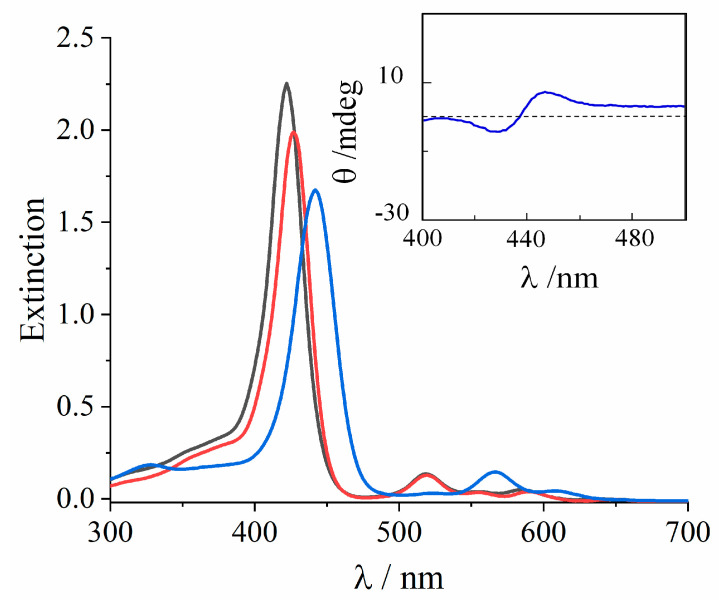
UV/Vis extinction spectra of TMpyP(4) (black line), TMpyP(4)/PGA (red line), and the end of the aggregation process promoted by the addition of Zn^2+^ (blue line). Experimental conditions: [TMpyP(4)] = 10 µM, [PGA] = 500 µM, [Zn^2+^] = 200 µM, PGA MW = 13,600, acetate buffer = 10 mM, pH = 4.5, and T = 298 K.

Figure 1 shows a comparison of the UV/Vis spectra for the neat TMpyP(4), its supramolecular adduct on PGA in a ratio of [PGA]/[TMpyP(4)] = 50, and that of ZnTMpyP(4) on PGA under the same experimental conditions ([TMpyP(4)] = 10 µM). The B-band of free TMpyP(4) was centered at 422 nm and, by adding PGA, it undergoes to a small red-shift and hypochromicity. This observation was expected for the interaction of the charged porphyrin on the surface of the polymer [20]. The metal derivative ZnTMpyP(4) displayed a further bathochromic shift to 442 nm. The pH of the solution was maintained at 4.45 in a 10 mM acetate buffer, ensuring that PGA was in an α-helical conformation [19].

**Figure 2:**
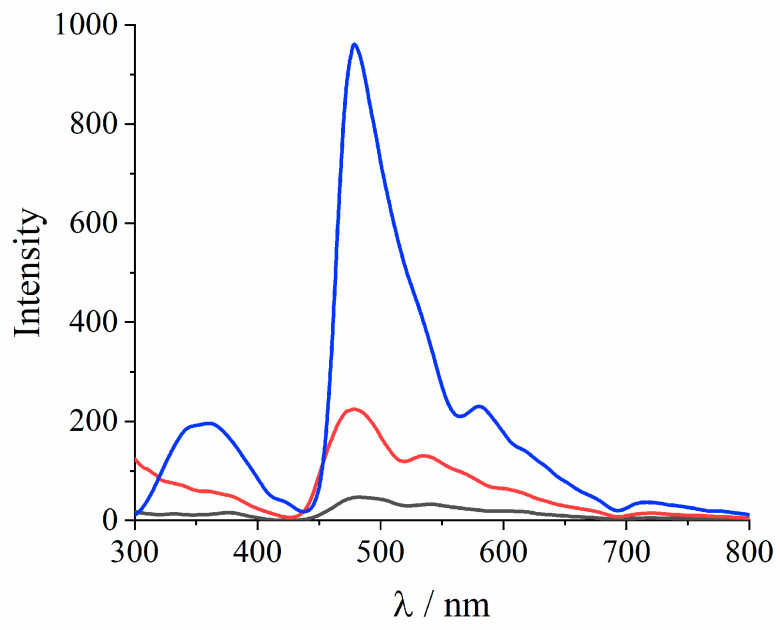
RLS spectra of TMpyP(4) (black line), TMpyP(4)/PGA (red line), and the end of the aggregation process promoted by the addition of Zn^2+^ (blue line). Experimental conditions: [TMpyP(4)] = 10 µM, [PGA] = 500 µM, [Zn^2+^] = 200 µM, PGA MW = 13,600, acetate buffer = 10 mM, pH = 4.5, and T = 298 K.

Under these conditions, chirality was transferred from the polymeric matrix to the chromophore assembly, as evidenced by the appearance of a moderately intense bisignate circular dichroism band centered at 442 nm (inset of Figure 1). The aggregated nature of this chromophore was confirmed by resonance light-scattering experiments (RLS, Figure 2) [21]. The RLS profile of the neat porphyrin revealed a deep well at 422 nm (Figure 2), in line with the monomeric state of this chromophore. The addition of PGA (Figure 2) led only to a moderate increase in the scattered light in the sample, indicating a partial aggregation or a weak electronic coupling among the porphyrins in the aggregate [22]. The final metal derivative on the polymer displayed a consistent increase in the RLS intensity (Figure 2), pointing to the formation of extended aggregates or to a stronger electronic coupling with respect to the un-metalated porphyrin.

**Figure 3:**
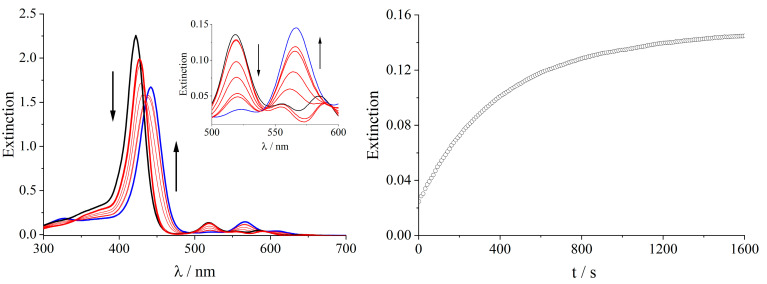
UV/Vis extinction spectral changes and relative magnification in the Q-band region (on the **left**) and corresponding kinetic profile at 566 nm for the formation of Zn(II)TMpyP(4) promoted by PGA (on the **right**). The arrows mark the decrease in the B-band relative to the starting TMpyP(4)/PGA adduct and the parallel increase in the B-band of the metal derivative on the polymer. Experimental conditions: [TMpyP(4)] = 10 µM, [PGA] = 500 µM, [Zn^2+^] = 200 µM, PGA MW = 13,600, acetate buffer = 10 mM, pH = 4.5, T = 298 K.

All the kinetic experiments were performed by monitoring the formation of the zinc(II) derivative of TMpyP(4) using the changes in absorbance in the UV/Vis absorption spectra. We selected 566 nm as the optimal wavelength, which corresponds to the Q-band of ZnTMpyP(4). This choice was dictated by the consistent overlap of the B-bands of TMpyP(4) and its metal derivative on the polymer (≈14 nm). Figure 3 reports a typical spectral change for the metal insertion in TMpyP(4) on PGA, together with the kinetic trace at the Q-band maximum. An inspection of these data revealed that the kinetics obeyed a first-order rate law, which can be easily fitted by a first-order model, resulting in the corresponding observed pseudo-first-order rate constant, *k*_obs_ (s^−1^).

In the absence of PGA and at an acidic pH, the rate law for metal insertion has the form: *k*_0_ = *k*_1_[Zn^2+^][X^−^]/{1+([H^+^]/K_3_) + ([H^+^]^2^/K_3_K_4_)}, according to the following mechanism [23]:

H_2_TMpyP(4)^2+^ ↔ HTMpyP(4)^+^ + H^+^ (1)

HTMpyP(4)^+^ ↔ TMpyP(4) + H^+^ (2)

TMpyP(4) + X^−^ + Zn^2+^ ↔ ZnTMpyP(4) (3)

In this model, it is assumed that (i) the free-base porphyrin TMpyP(4) can be mono-protonated (HTMpyP(4)^+^) and diprotonated (H_2_TMpyP(4)^2+^) via Equilibria 1 and 2 and (ii) the only reactive species is TMpyP(4). The values reported in the literature for the above equilibria are pK_3_ = 2.06 ± 0.5 and pK_4_ = 0.8 ± 0.1, thus ensuring that, for a pH higher than 3, only the free base is present in the solution.

**Figure 4:**
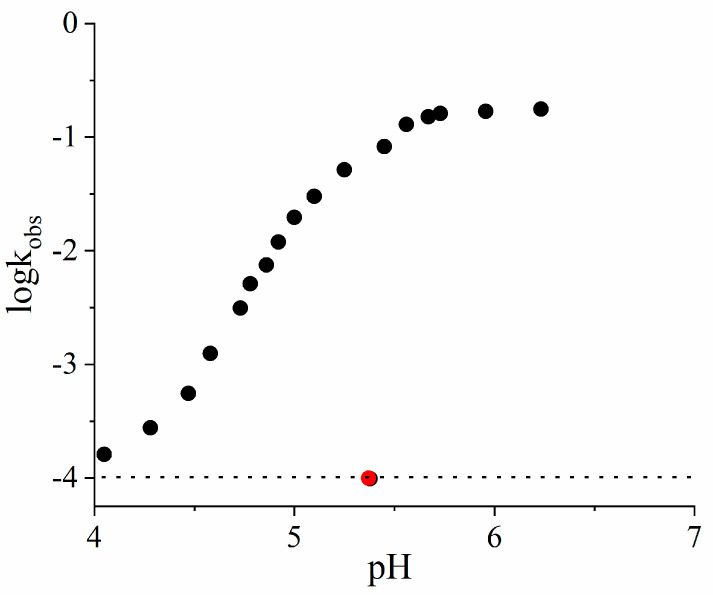
Logarithmic plot of the pseudo-first-order rate *k_obs_* (s^−1^) for the formation of Zn(II)TMpyP(4) promoted by PGA as a function of pH. Measurements conducted at pH values above 5.6 were carried out by employing a citrate buffer at the same ionic strength value (10 mM). The red point is the value of the *k_obs_* measured for the reaction of Zn^2+^ with TMpyP(4) in the absence of PGA, at a pH of 5.38. Experimental conditions: [TMpyP(4)] = 10 µM, [PGA] = 500 µM, [Zn^2+^] = 200 µM, PGA MW = 13.600, acetate buffer = 10 mM, and T = 298 K.

Under the ratio [PGA]/[TMpyP(4)] = 50, the metalation kinetics were investigated by varying the pH in the range of 3–6. The quite-large value of the relative concentration of porphyrin and PGA was chosen to ensure that, even when PGA was at lower pH values, favorable electrostatic contacts would be effective between negatively charged carboxylate residues and the tetra-cationic TMpyP(4). Actually, this ratio seemed to be the best compromise to preserve the colloidal stability of the supramolecular adduct, retaining a strong accelerating effect on the rates. Higher PGA loads contributed to the distribution of the porphyrin in a more dispersed fashion, thus lowering its metalation rate. Figure 4 and Table 1 report the dependence of *log k*_obs_ on the pH, which showed a sigmoidal profile with an inflection point at a pH of 4.7. This value is very close to the pH value for the transition of the PGA scaffold from an α-helix (at an acidic pH) to a random coil (at a neutral pH) [19].

It was observed that the metalation rates increased with increasing pH. The catalysis process was largely enhanced at pH values that stabilized the random coil conformation of PGA. The transition from the coiled α-helix to this more open conformation was due to the deprotonation of the carboxylic groups on the side chains of the amino acids. Consequently, the density of negative charges around the polymer scaffold was much higher with respect to the α-helix. A comparison of the values for the rate constants measured around a pH of 5.4 in the absence (*k_obs_* = (9.86 ± 0.05) × 10^−5^ s^−1^) and presence (*k_obs_* = (7.94 ± 0.09) × 10^−2^ s^−1^) of the biopolymer showed that PGA accelerated the insertion of the metal ion by almost three orders of magnitude.

Therefore, metalation was promoted by the matrix, which facilitated porphyrin aggregation through the electrostatic interactions between the negatively charged carboxylate groups and the positively charged N-methyl pyridyl moieties of TMpyP(4). Furthermore, the electrostatic field exerted by the biopolymer promoted the condensation of Zn^2+^ ions in close proximity to the porphyrin, thus facilitating an encounter between the reactants and lowering the activation barrier for metal coordination into the macrocycle.

**Figure 5:**
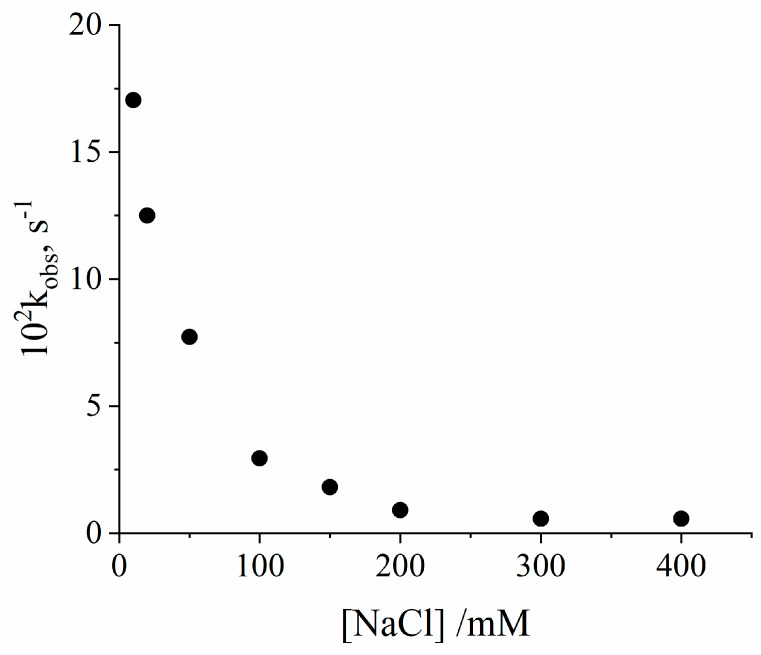
Plot of the pseudo-first-order rate *k_obs_* (s^−1^) for the formation of Zn(II)TMpyP(4) promoted by PGA as a function of ionic strength at a constant pH. Experimental conditions: [TMpyP(4)] = 10 µM, [PGA] = 500 µM, [Zn^2+^] = 200 µM, PGA MW = 13,600, acetate buffer = 10 mM, pH = 5.6, and T = 298 K.

In order to obtain more insights into the role of PGA in this process, we investigated the effect of the addition of NaCl to the system. Figure 5 shows that the observed rate constant *k_obs_* decreased when increasing the ionic strength of the solution. This evidence can be explained in terms of the progressive reduction in the electrostatic interactions between the negatively charged carboxylate groups and both the porphyrins and the metal ions. A similar ionic strength effect on the stability of self-assembled porphyrins on a highly charged polymeric support has already been reported for the interaction of TMpyP(4) and poly(vinylsufonate) [24].

At a pH of 5.6, PGA can be considered in its highly charged random coil conformation. Our experimental findings can be explained by the change in the electrostatic potential around the polymer according to the Boltzmann–Poisson theory. At large distances from the polyelectrolyte, typically greater than Debye’s length, the far-field behavior of the electrostatic potential had a tendency to exponentially decrease in accordance with the solutions of the equations for a cylinder [25,26], and its value could be modulated by varying the ionic strength of the medium. It is interesting to note that an increase in the concentration of acetate anions in the buffer led to a decrease in the reaction rates (*k_obs_* = (1.29 ± 0.08) × 10^−1^ s^−1^ at [CH_3_COO^−^] = 10 mM; *k_obs_* = (4.87 ± 0.04) × 10^−3^ s^−1^ at [CH_3_COO^−^] = 300 mM, pH of 5.6). The rate law determined by Hambright [23] displayed a first-order dependence on the concentration of the added anions. In our case, our experimental results suggested that the acetate simply contributed to the total ionic strength, thus screening for the attractive interactions among the reagents.

## 3. Materials and Methods

### 3.1. Materials

The 5,10,15,20-tetrakis-(N-methylpyridinium-4-yl)porphyrin (TMpyP(4)) was purchased from Sigma-Aldrich as a chloride salt and utilized as received. A stock solution of porphyrin (≈200 μM) was freshly prepared by dissolving the solid in dust-free water (Galenica Senese) and stored in the dark in glass or PMMA vials to avoid photodegradation. The solution concentrations were determined using UV/Vis from the known molar extinction coefficient at the Soret maximum (TMPyP(4): 2.34 × 10^5^ M^−1^ cm^−1^, λ = 422 nm) [27]. The sodium salt of poly-L-glutamic acid (MW~13,600 kD) was purchased from Aldrich Chemicals Co. A stock solution of the polypeptide was prepared by dissolving the solid in an acetate buffer at a pH of 4.5 (ionic strength of 5 mM). The concentrations of the peptide solution were determined spectrophotometrically, using ε_205_ = 2150 M^−1^ cm^−1^ [28]. Zinc acetate and all the other reagents were supplied by Aldrich Chemicals Co. and used without further purification. All glassware and cells employed in the experiments were first cleaned with concentrated HNO_3_, H_2_SO_4_, or an acidic piranha mixture (H_2_O_2_/H_2_SO_4_ 1:3 *v*/*v*), followed by cleaning with standard ionic detergents, and were eventually rinsed with deionized and HPLC-grade water.

### 3.2. Methods

UV/Vis absorption spectra and kinetic traces were acquired using an Agilent model 8453 diode-array spectrophotometer with 1 cm path-length quartz cells (Hellma). A UV filter (Hoya glass type UV-34, cut-off: 340 nm) interposed between the lamp and the samples was utilized in the measurements to cut off the UV component of the spectrophotometer lamp, preventing the photodegradation of the porphyrin samples. Resonance light-scattering (RLS) experiments were performed on a Jasco model FP-750 spectrofluorometer by adopting a synchronous scan protocol of both excitation and emission monochromators with a right-angle geometry [21]. The circular dichroism (CD) spectra were recorded on a JASCO J-710 spectropolarimeter equipped with a 450 W xenon lamp. The CD spectra were corrected for both the cell and solvent contributions. All the kinetic experiments were started by adding a known amount of an aqueous solution of the Zn(II) ion to a premixed solution of porphyrin and polypeptides in the proper concentration ratio required by the experiment, and then the mixture was inverted three times to ensure the mixing of the reactants. Kinetic runs were carried out in the thermostatic compartment of the spectrophotometer, with a temperature accuracy of ±0.1 K. The analyses of the kinetic profiles acquired at 566 nm were performed using a non-linear fit of the extinction data according to a first-order reaction (Equation (1)), providing the observed rate constant (*k_obs_*, s^−1^).

Ext*_t_* = Ext_f_ + (Ext_0_ − Ext_f_) exp(−*k_obs_t*) (4)

where Ext_0_, Ext_f_, and *k_obs_* are the parameters to be optimized (Ext_0_ = initial extinction at *t* = 0 soon after mixing reagents; Ext*_t_* = extinction at time *t*; and Ext_f_ = final extinction at the end of the reaction).

## 4. Conclusions

When in close proximity to charged surfaces, counter-ion condensation is responsible for interesting catalytic effects on the metalation of porphyrins. PGA is a versatile model system that can provide a way to deeply investigate changes in reactivity. This biopolymer undergoes a conformational change from a random coil to an α-helix upon the progressive protonation of the side chains on the constituent amino acid residues. The changes in the overall charge and in the rigidity of the backbone were responsible for the interactions of species bearing opposite charges and with different steric demands, such as meso-substituted porphyrins and simple metal ions. Our experimental evidence points to a consistent enhancement of the metal insertion rates when TMpyP(4) was bound to the biopolymer. This effect outlines the importance of these scaffolds in various applications where reaction rates need to be accelerated, e.g., in sensing. In addition, from a fundamental point of view, it gives insights into the reactivity of porphyrins in biologically relevant environments.

## Figures and Tables

**Table 1:** Pseudo-first-order rate *k_obs_* (s^−1^) for the formation of Zn(II)TMpyP(4) promoted by PGA as a function of pH. Experimental conditions: [TMpyP(4)] = 10 µM, [PGA] = 500 µM, [Zn^2+^] = 200 µM, PGA MW = 13,600, acetate buffer = 10 mM, and T = 298 K.

10^5^ [H^+^]/M	10^3^ *k_obs_*/s^−1 a^
8.91	0.16 ± 0.01
5.24	0.28 ± 0.02
3.34	0.55 ± 0.04
2.63	1.24 ± 0.01
1.86	3.12 ± 0.03
1.66	5.10 ± 0.06
1.39	7.48 ± 0.04
1.20	11.90 ± 0.05
1.00	19.60 ± 0.06
0.79	30.00 ± 0.01
0.56	51.50 ± 0.03
0.35	82.40 ± 0.08
0.27	129 ± 0.009
0.21	151 ± 0.004
0.18	161 ± 0.003
0.11	169 ± 0.007
0.06	176 ± 0.008
0.42 ^b^	0.098 ± 0.002

^a^ From a non-linear least-squares fit of the experimental extinction data at 566 nm (see Experimental Section). ^b^ Value associated with the non-catalytic process in the absence of PGA, at a pH of 5.38.

## Data Availability

The data are available upon request.
